# XPS analysis of polymer brush coatings

**DOI:** 10.1039/d6py00562d

**Published:** 2026-08-03

**Authors:** Andriy R. Kuzmyn, Gerrit-Jan Linker, Yibin Bu, Sissi de Beer

**Affiliations:** a Department of Molecules & Materials, MESA+ Institute, University of Twente 7500AE Enschede The Netherlands andriy.kuzmyn@gmail.com a.r.kuzmyn@utwente.nl s.j.a.debeer@utwente.nl; b MESA+ Institute for Nanotechnology, University of Twente 7522 NB Enschede The Netherlands

## Abstract

X-ray photoelectron spectroscopy (XPS) is a powerful surface-sensitive technique widely used for the chemical characterization of thin films and interfaces. XPS detects the outermost ∼10 nm of organic films, making it useful for studying the surface of polymer-brush coatings. Despite the widespread use of XPS for polymer brushes, data interpretation and reporting practices often remain inconsistent, particularly for chemically complex or beam-sensitive systems. This tutorial review provides a practical overview of XPS analysis of polymer-brush coatings, with emphasis on experimental considerations, spectral interpretation, and common pitfalls. Key aspects of sample preparation are discussed, including contamination control, charge compensation, and strategies to minimize beam-induced degradation in radiation-sensitive polymers. The interpretation of survey spectra is examined with a focus on elemental quantification, background selection, and complications arising from spin–orbit splitting. Strategies for estimating polymer-brush thickness from substrate signal attenuation are outlined together with the assumptions and limitations inherent to attenuation-based analysis. Detailed guidance is provided for high-resolution C 1s analysis, including background modeling, chemically consistent peak fitting, and assignment of common carbon environments, while highlighting common overfitting artifacts. The complementary role of density functional theory (DFT) calculations in supporting chemically meaningful peak assignment and validating spectral interpretation is also discussed. By combining practical recommendations with critical discussion of common sources of error, this tutorial aims to support more reliable and reproducible XPS characterization of polymer brush coatings.

## Abstract 2

XPS examines the near-surface region of organic films. This tutorial covers XPS analysis of polymer-brush coatings: sample preparation, charge referencing, survey quantification, C 1s fitting, depth profiling, and DFT-assisted peak assignment.

## Graphical abstract

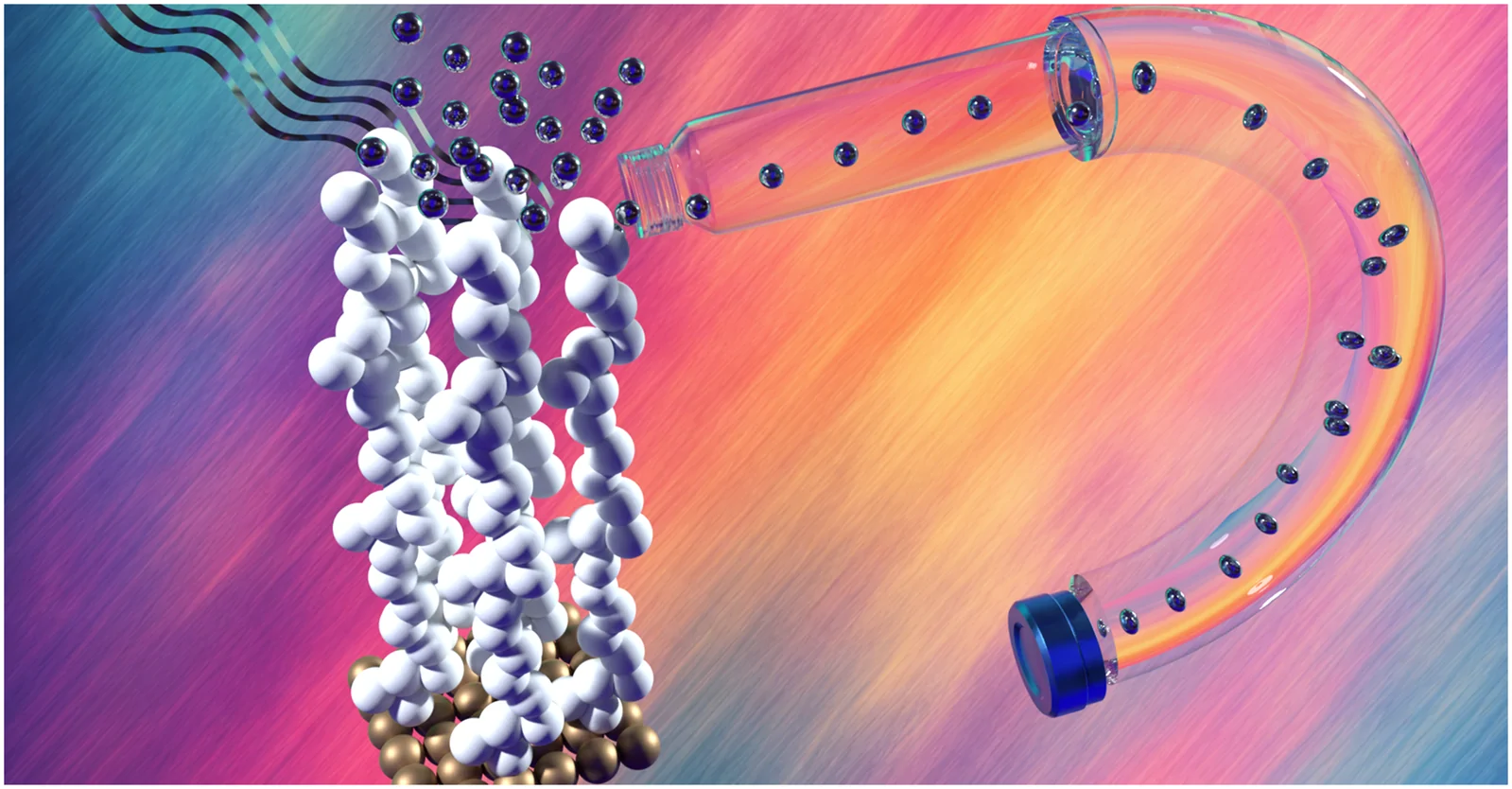

## Introduction

X-ray photoelectron spectroscopy (XPS), also known as electron spectroscopy for chemical analysis (ESCA), is one of the most widely used surface-sensitive techniques for probing the elemental composition and chemical states of solid surfaces and thin films.^1,2^ Because photoelectrons that escape without energy loss originate only from the top few nanometers of a material, as determined by the inelastic mean free path of the photoelectrons and the emission geometry, XPS provides inherently surface-sensitive information.^3,4^ The technique enables quantitative elemental and chemical-state characterization and can be applied to monitor chemical and electrochemical reactions occurring at surfaces and interfaces.^1,2,5–9^ With the exception of hydrogen and helium, whose photoionization cross-sections are negligible, all elements from lithium to uranium are detectable by XPS. In the context of polymer brushes, this means that essentially every heteroatom of interest can be identified and quantified, including N, O, S, P, F, Cl, Br and Si, as well as metal centers in the substrate or in metal-containing side groups.

Polymer brushes are well suited for XPS: for thin coatings the signal contains contributions from the entire film, including the substrate and the grafting interface, whereas for thicker coatings it originates almost entirely from the outer brush surface. Polymer brushes consist of densely tethered polymer chains end-grafted to a surface, commonly prepared using surface-initiated controlled radical polymerization methods such as surface-initiated atom transfer radical polymerization (SI-ATRP), surface-initiated reversible addition–fragmentation chain-transfer polymerization (SI-RAFT), single-electron-transfer living radical polymerization (SET-LRP), or surface-initiated photoinduced electron-transfer RAFT polymerization (SI-PET-RAFT).^9–20^ Owing to their high grafting density and tunable chemical composition, polymer brushes exhibit a wide range of functional interfacial properties, including antifouling behavior,^22,23^ lubrication,^24–30^ antiviral activity,^31^ sensing capabilities,^21,32–36^ controlling protein adhesion and release,^37^ and control over biointeractions at surfaces.^38,39^ As a result, reliable chemical characterization of these thin interfacial layers is essential for understanding structure–property relationships and validating surface functionalization strategies.

Complementary surface-characterization techniques such as Fourier-transform infrared (FTIR),^40,41^ Raman spectroscopy,^42,43^ spectroscopic ellipsometry, and scanning electron microscopy combined with energy-dispersive X-ray spectroscopy (SEM-EDS)^44^ are also frequently used for polymer brush analysis. However, XPS offers a unique combination of surface sensitivity, elemental quantification, and chemical-state information, making it particularly valuable for ultrathin polymer coatings and interfacial functionalization studies.

Despite the widespread use of XPS for polymer-brush characterization, reliable interpretation of spectra requires careful consideration of experimental artifacts and data-analysis procedures.^45^ Surface contamination, differential charging, overlapping C 1s components, beam-induced degradation, and inconsistent fitting methodologies can all complicate chemical-state assignment and quantitative interpretation.^45–47^ In addition, quantitative interpretation of XPS data, including thickness estimation from substrate signal attenuation and depth profiling of layered brushes, requires careful consideration of the assumptions and limitations inherent to these approaches.^48,49^ Reporting practices and fitting methodologies also vary substantially between studies, complicating comparison and reproducibility across the literature.

**Scheme 1:**
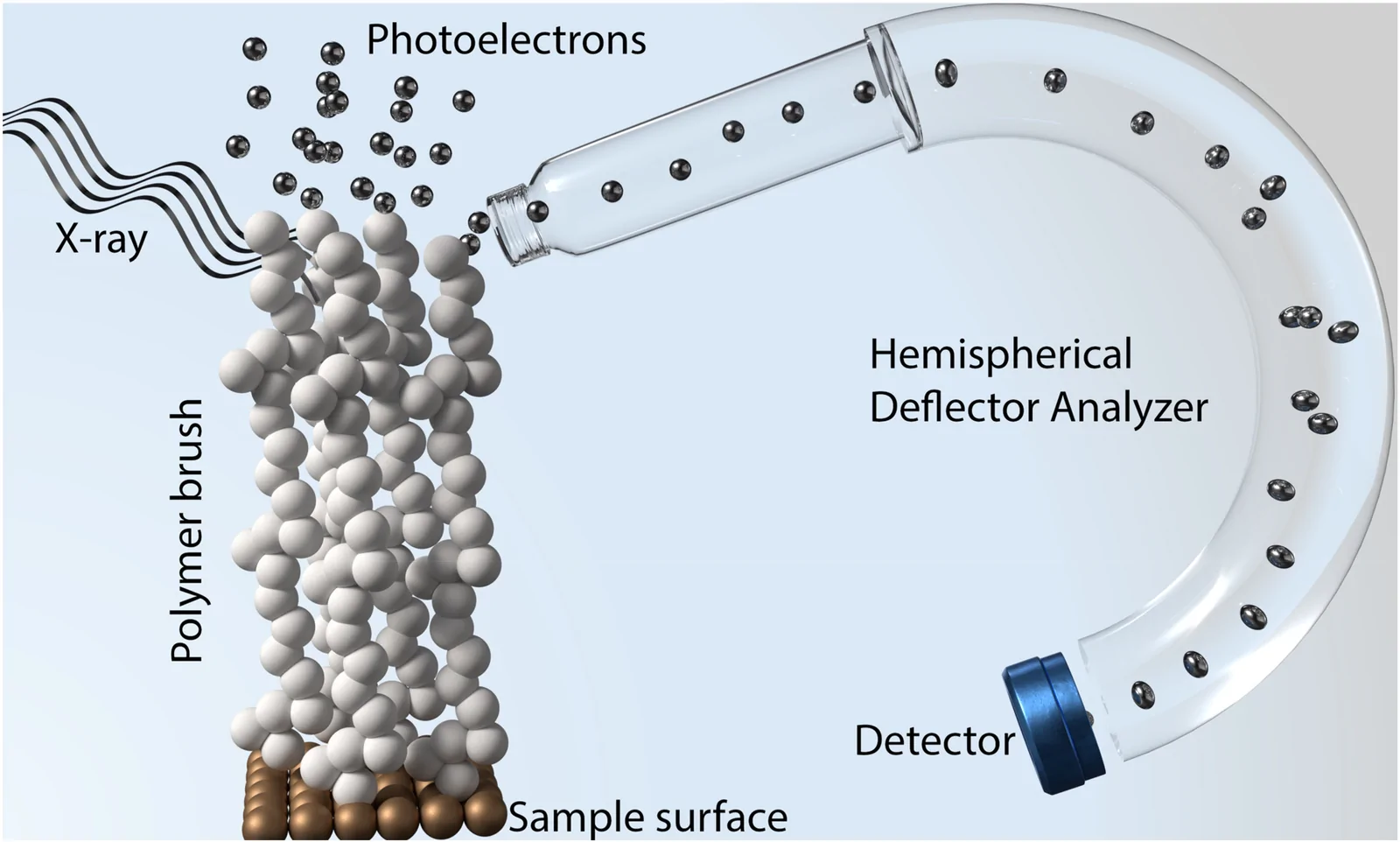
Schematic illustration of the X-ray photoelectron spectroscopy (XPS) analysis of a polymer brush coating. A monochromated Al Kα X-ray beam irradiates the sample surface, inducing the emission of photoelectrons from the polymer chains. The emitted electrons are collected by a hemispherical energy analyzer, which measures their kinetic energies.

The physical principle underlying X-ray photoelectron spectroscopy (XPS) is the photoelectric effect, first observed by Heinrich Rudolf Hertz in 1887 and theoretically explained by Albert Einstein in 1905, work for which Einstein was awarded the 1921 Nobel Prize in Physics.^50,51^ In XPS, monochromatic X-rays irradiate a material and induce the emission of core-level electrons.^52^ By measuring the kinetic energy of these photoelectrons, their binding energies can be determined, enabling identification of the elements present, their chemical states, and the local electronic environments in which they reside. Shortly after Einstein's work, P. D. Innes recorded one of the first X-ray-induced electron spectra in 1907 using a Röntgen tube and magnetic analyzer, thereby demonstrating the fundamental principle underlying modern XPS.^53^ The modern form of XPS was established through the pioneering work of Kai Siegbahn and co-workers, whose development of high-resolution photoelectron spectroscopy laid the foundation for modern surface analysis.^54^ (Scheme 1) Since then, XPS has become a standard technique in materials science, surface chemistry, polymer science, and nanotechnology.

In this tutorial article, we provide a practical overview of XPS analysis of polymer brush coatings, with emphasis on experimental considerations, spectral interpretation, and common pitfalls encountered in routine analysis. Particular attention is given to sample preparation, contamination control, charge compensation, survey-spectrum interpretation, chemically consistent C 1s peak fitting, attenuation-based thickness estimation, and depth profiling using gas cluster ion beams. The complementary role of density functional theory (DFT) calculations in supporting chemically meaningful peak assignment and interpretation of XPS spectra is also discussed. The aim of this tutorial is not only to summarize commonly used approaches but also to highlight important limitations, sources of error, and best practices relevant to reliable and reproducible XPS characterization of polymer brush coatings.

## Practical applications of XPS in polymer brush research

Before discussing measurement procedures, spectral interpretation, and peak fitting strategies, it is important to consider what information XPS can provide for polymer brush coatings. Beyond the obvious determination of elemental composition and detection of surface contaminants, XPS is widely used throughout polymer-brush synthesis workflows to monitor and verify individual chemical modification steps. In addition to identifying adventitious contamination, XPS can detect residual species originating from brush synthesis and processing, including solvents, catalyst residues, ligands, initiator fragments, unreacted monomers, and trace metal contaminants.

XPS is particularly valuable for confirming successful surface functionalization during the preparation of anchoring and initiator layers. For example, the immobilization of brominated initiators for surface-initiated atom transfer radical polymerization (SI-ATRP) or sulfur-containing RAFT agents can be verified by analyzing Br 3d, Cl 2p, S 2p, or related core-level signals. In some cases, XPS can also provide qualitative or semi-quantitative information on initiator surface density, for example, by comparing halogen or sulfur signals relative to substrate peaks or to mixed self-assembled monolayers containing inert spacer molecules. However, interpreting such measurements requires caution because halogen- and sulfur-containing species may undergo beam-induced degradation or partial loss during XPS analysis, as discussed in later sections.

Following polymerization, XPS can be used to confirm successful brush growth through changes in elemental composition, attenuation of substrate signals, and appearance of characteristic chemical functionalities associated with the polymer structure. While attenuation-based analysis can provide estimates of effective film thickness, these values depend strongly on assumptions about film homogeneity, density, and electron attenuation lengths and should therefore be interpreted with caution. These aspects are discussed in the Wide XPS spectra section in detail.

XPS is also widely used to evaluate post-polymerization modification reactions, including side-chain functionalization, biomolecule immobilization, click reactions, quaternization, and incorporation of responsive or bioactive groups.^55^ Furthermore, XPS depth profiling using gas cluster ion beams (GCIB) can provide insight into compositional gradients and multilayer architectures within polymer brushes, enabling verification of diblock brush structures, buried interfaces, and surface-enriched functional layers. Representative examples of these applications are summarized in Table 1.

**Table 1:** Representative applications of XPS in polymer-brush synthesis, modification, and characterization

Category	What XPS reveals
Initiator immobilization and initiator density	Confirmation of initiator attachment, bromine/chlorine density, mixed initiator layers, surface coverage, initiator monolayer attenuation-based thickness estimation^33,37,56–58^
Polymer-brush composition and film formation	Confirmation of polymer growth, monomer incorporation, elemental ratios, and attenuation-based thickness estimation^18,31,32,36–38,56,59–63^
Post-polymerization modification and end-group analysis	Surface functionalization, biomolecule attachment, side-chain modification, RAFT/ATRP end-group analysis, and transformation^19,64–66^
Depth profiling	Layered structures, compositional gradients, buried interfaces, sequential block growth^19,60,62^
Surface contamination and beam-induced degradation	PTFE contamination, adventitious carbon, X-ray damage, loss of functional groups^67^

## XPS measurement

Reliable X-ray photoelectron spectroscopy (XPS) analysis of polymer brush coatings requires careful attention to sample preparation, acquisition conditions, charge compensation, and data-processing procedures.^1,68^ Because polymer brushes are thin, chemically complex, and often sensitive to X-ray irradiation, experimental artifacts can strongly influence spectral quality, peak positions, and quantitative interpretation.^45,48,67^ The following section outlines practical considerations and common pitfalls associated with XPS analysis of polymer brush systems (please also see **A practical workflow for XPS of polymer brushes below**).

### Sample preparation and contamination control

Prior to XPS analysis, polymer-brush samples should be thoroughly dried to remove residual solvent and adsorbed moisture. Polymer brushes are commonly synthesized *via* surface-initiated controlled radical polymerization and may retain residual catalyst species, ligands, unreacted monomers, physisorbed oligomers, or solvent impurities after synthesis. Thorough washing and purification prior to XPS analysis are therefore essential, since even trace residual contaminants can strongly influence survey spectra and high-resolution peak fitting. In addition, the long-term chemical stability of polymer brushes depends strongly on the nature of the anchoring chemistry and storage conditions.^69^ For example, silane-based anchoring layers on silicon or oxide substrates may undergo hydrolysis, rearrangement, or partial degradation upon prolonged exposure to moisture, solvents, or elevated temperatures,^70,71^ whereas thiol-based self-assembled monolayers on gold surfaces are susceptible to oxidation and desorption over time.^69^ Consequently, prolonged storage prior to XPS analysis should generally be avoided, particularly under ambient conditions. When possible, samples should be stored under an inert atmosphere, in dry conditions, and protected from prolonged light exposure in order to minimize surface oxidation, hydrolysis, and other degradation processes that may alter the measured surface composition. Since XPS is inherently surface-sensitive, even trace contamination can significantly influence the measured composition and chemical-state distribution.^1,68,72^ Surface contamination most commonly arises from adventitious hydrocarbons, dust particles, residual solvents, or handling during sample preparation and transfer.^68,72^ Samples should therefore be handled in a clean environment and, when appropriate, gently blown with inert gas (*e.g.*, nitrogen) to remove particulate contamination before introduction into the spectrometer.

Polymer brushes may also undergo structural changes upon drying and transfer into ultrahigh vacuum.^73,74^ In particular, swollen brush conformations present under ambient or liquid conditions may partially collapse in vacuum, potentially altering film density and attenuation behavior during XPS measurements. Consequently, XPS measurements generally reflect the dry-state structure of the polymer brush rather than its solvated morphology.

A sample size of approximately 1 cm × 1 cm is typically convenient for mounting and handling; however, many modern XPS instruments employ focused X-ray beams with spot sizes ranging from several hundred micrometers down to below 10 µm. As a result, considerably smaller samples may also be analyzed, provided the measured region is representative of the coating. The acceptable sample dimensions depend strongly on the instrument geometry, sample holder design, and vacuum chamber configuration. Depending on the instrument, samples ranging from approximately 1 mm × 1 mm up to 10 cm × 10 cm or larger may be accommodated for XPS analysis. Because polymer brushes may exhibit lateral heterogeneity arising from incomplete grafting, defects, dust contamination, or surface microstructures, spectra should ideally be acquired from multiple regions of the surface to assess sample homogeneity.

Particular care should be taken to avoid fluorine contamination originating from PTFE-coated (Teflon) tweezers, seals, containers, or gloves. Owing to the high relative sensitivity factor (RSF) of fluorine, even trace contamination can generate pronounced F 1s (∼688–689 eV) signals that may obscure or complicate the interpretation of the true surface composition.^75,76^ This caution concerns adventitious fluorine rather than fluorine-containing brushes themselves: fluorinated polymers are readily, and very sensitively, characterized by XPS (Table 5), and the same high sensitivity factor that makes contamination so conspicuous also makes fluorine an excellent marker for deliberately fluorinated systems.

### X-ray source and acquisition parameters

Most laboratory XPS measurements of polymer brush coatings are performed using monochromated Al Kα radiation (photon energy 1486.6 eV), which provides a suitable balance between spectral resolution and signal intensity for organic materials.^52,77^ Depending on the instrument configuration, selectable X-ray spot sizes typically range from several hundred micrometers to below 10 µm for scanning microprobe systems. When analyzing patterned or spatially heterogeneous surfaces, the relationship between feature dimensions and the effective analysis area should be carefully considered,^60,78,79^ since spectra may otherwise contain contributions from both the target structure and surrounding regions.

**A practical workflow for XPS of polymer brushes**:

**1. Sample handling and storage:** dry the sample, avoid PTFE contact (fluorine), store in a clean, inert container; mount and blow off particulates.

**2. Low-dose survey:** record a first survey at reduced dose to identify all elements before prolonged exposure.

**3. Contaminants and substrate:** check for adventitious C, fluorine, and residual substrate signal; decide whether substrate visibility reflects thin film or incomplete coverage.

**4. High-resolution scans of diagnostic regions:** acquire the beam-sensitive regions (N 1s, S 2p, Br 3d, Cl 2p) first, then C 1s.

**5. Charge correction and referencing:** reference to a conductive substrate peak where visible; otherwise to C–C/C–H at 285.0 eV, consistently, reporting raw and corrected BEs where positions matter.

**6. Background and constraints:** choose Shirley (narrow, flat) or Tougaard (wide, strong loss tails); constrain component number, FWHM, and doublet ratios to the chemistry.

**7. Quantification and stoichiometric check:** use sensitivity-factor-corrected areas; compare the measured composition against the monomer stoichiometry.

**8. Beam-damage control:** monitor repeated scans; move to a fresh spot for final spectra.

**9. Optional angle-resolved XPS or GCIB depth profiling:** for depth information; report cluster and crater parameters.

**10. Minimum reporting**: record all acquisition and processing parameters (Table 2).

**Table 2:** Minimum information to report for an XPS measurement

Category	Parameters to report
Instrument/source	X-ray source and energy (*e.g.* monochromated Al Kα, 1486.6 eV); spot size; power
Acquisition	Pass energy (survey and high-res); step size; dwell time; number of scans; take-off/emission angle
Charge control	Neutralizer type and settings; whether flood gun on/off; post-acquisition charge-correction method
Energy calibration	Reference used (C–C/C–H 285.0 eV, Au 4f_7/2_, Si 2p…); raw and corrected BEs where relevant
Processing	Software and version; background model (linear/Shirley/Tougaard); line shape and convention (*e.g.* GL(30)); FWHM constraints; doublet constraints
Quantification	RSF set/library used; whether transmission correction applied
Damage test	Whether repeated-scan damage tests were performed and their outcome

Acquisition parameters such as analyzer pass energy, step size, dwell time, and number of scans strongly influence spectral quality and quantitative reliability.^1,68^ In general, lower pass energies provide improved energy resolution and facilitate separation of closely spaced chemical states, particularly in complex C 1s envelopes, but at the expense of reduced count rate and increased acquisition time. Conversely, higher pass energies improve signal intensity and are commonly used for survey spectra where ultimate spectral resolution is less critical. Typical pass energies are approximately 50–200 eV for survey scans and 10–40 eV for high-resolution spectra, although the optimal settings depend on the instrument and material system.

To ensure reproducibility and facilitate comparison between studies, XPS measurements should be reported together with key acquisition and processing parameters. Important experimental details include the X-ray source, spot size, analyzer settings, take-off angle, charge neutralization conditions, calibration procedure, background model, peak-shape functions, and fitting constraints. Reporting these parameters is particularly important for polymer brush systems, where small variations in acquisition conditions may significantly influence peak positions, line shapes, and quantitative interpretation.

### Surface sensitivity

Three related but distinct depth quantities govern XPS of thin organic films. The inelastic mean free path (*λ*) is the average distance a photoelectron travels between inelastic collisions; for C 1s photoelectrons excited by Al Kα (kinetic energy ≈ 1200 eV) it is approximately 3–3.5 nm in typical polymers.^3^ The effective attenuation length (*L*) also accounts for elastic scattering; it is somewhat smaller than *λ* (typically by ∼10–20% for organic overlayers) and is the quantity to be used in overlayer-attenuation equations.^4^ The information depth, commonly taken as 3*λ* cos *θ*, the depth above which ∼95% of the detected signal originates, where *θ* is the emission angle from the surface normal, is therefore about 10 nm for polymers at normal emission and decreases at grazing emission.^1,80^ None of these is a hard cut-off: the signal decays exponentially with depth, so XPS always weights the outermost layers most strongly. Consequently, a substrate signal may sometimes remain detectable even when the nominal brush thickness exceeds the information depth. Its visibility depends on factors such as brush grafting density, film uniformity and roughness, the presence of low-density regions or defects, and the relative XPS signal strength of the substrate. A detectable substrate signal should therefore not be interpreted solely as evidence that the brush is thinner than the nominal information depth.

### Charge compensation and energy referencing

Because polymer brush coatings are typically electrically insulating, positive surface charging frequently occurs during XPS analysis. Differential charging can lead to peak broadening, asymmetric line shapes (for example, peaks exhibiting tailing or skewing toward higher binding energy), and shifts in apparent binding energy, thereby complicating chemical-state assignment and quantitative analysis. To minimize these effects, charge neutralization systems such as low-energy electron flood guns, ion compensation systems, or dual-mode neutralizers are commonly employed.^81^

The choice of charge compensation settings can substantially influence both spectral quality and beam-induced degradation behavior. Consequently, neutralizer conditions should be explicitly reported together with any post-acquisition charge correction procedures applied during data processing.

Binding energies of polymer-brush samples are commonly charge-corrected by setting the lowest-binding-energy C 1s component, assigned to C–C/C–H carbon, to 285.0 eV; values between 284.8 and 285.0 eV are also widely used. For carbon-rich brushes, particularly when no reliable external reference is available, this remains a practical approach. However, it should be treated as an approximate charge correction rather than an absolute calibration. When a conductive substrate signal is visible, such as Au 4f or Si 2p, referencing to that peak may provide a more robust alternative. Adventitious carbon is a less reliable reference because its apparent binding energy can vary with the substrate, surface condition, and charging behavior. Carbon referencing may also be unsuitable when the polymer lacks a clearly defined hydrocarbon C–C/C–H component. In heterogeneous brush–substrate systems, differential charging can shift regions or chemical components by different amounts, and such effects cannot be corrected by applying a single rigid shift across the entire spectrum. Moreover, forcing a C 1s component to exactly 285.0 eV may conceal genuine chemical shifts. Neutralization and referencing procedures should therefore be applied consistently and reported clearly, and both raw and corrected binding energies should be provided when absolute peak positions or small chemical shifts are important.

### Beam-induced degradation

Although XPS is often described as a non-destructive technique, prolonged X-ray exposure can induce chemical degradation in many polymeric materials. Beam damage is particularly relevant for polymer brushes because of their low thickness, limited material volume, and high concentration of radiation-sensitive functional groups. Degradation may result in changes in elemental composition, peak intensities, line shapes, and apparent film thickness during measurement.

Halogen-containing end groups, quaternary ammonium species, sulfur-containing functionalities, and other highly functionalized moieties are especially susceptible to X-ray-induced decomposition.^48,67^ Such degradation commonly arises from X-ray- or electron-induced bond cleavage, radical formation, desorption of labile fragments, and secondary electron damage pathways, which can progressively alter the chemical composition of the polymer surface during measurement. For example, polymer brushes containing quaternary ammonium groups have been reported to undergo rapid degradation during XPS analysis, manifested by reduction of charged nitrogen species, growth of hydrocarbon-like carbon contributions, and measurable thinning of the polymer layer.^48,67^ In some cases, degradation may be accelerated by charge compensation systems, likely due to electron-stimulated decomposition pathways.^45,48^

Beam-induced damage should be minimized and, where possible, actively monitored. Total acquisition time and repeated exposure of the same analysis area should be limited, particularly for brushes containing beam-sensitive groups such as quaternary-ammonium nitrogen, sulfur, bromine, or chlorine. A useful control is to acquire several short scans from the same region and compare the first and last scans for changes in peak area, position, line shape, or full width at half maximum before averaging. Sensitive regions, such as N 1s, S 2p, Br 3d, and Cl 2p, should preferably be measured early, after only a brief survey scan, and final quantitative spectra should be collected from a fresh, previously unexposed area when possible. The X-ray power, spot size, exposure time, and neutralizer settings should also be reported because they influence the delivered dose. Since electron neutralization may itself contribute to degradation, suitable control measurements should be used, where feasible, to distinguish X-ray-induced changes from electron-flood-gun effects.

Overall, reliable XPS characterization of polymer brush coatings requires careful consideration of contamination, charging, irradiation effects, and acquisition parameters. Because many of these factors can strongly influence spectral interpretation and quantitative analysis, consistent experimental procedures and transparent reporting practices are essential for obtaining reproducible and chemically meaningful results.^1,47,68^

## Wide XPS spectra

The survey or wide scan in XPS provides an overview of the elemental composition of polymer-brush coatings. For brushes of sufficient thickness (for example >20 nm) the measured elemental ratios (*e.g.*, C, N, O, S, Br, P) will often reflect the monomer unit composition of the polymer chain, provided that the underlying initiator or RAFT agent layer (on the substrate) does not significantly contribute to the detected signal. Although XPS is performed under high vacuum, a small contribution from atmospheric contaminants or adventitious carbon will always be present, which may slightly alter the overall C : N : O ratios.^72^

**Fig. 1:**
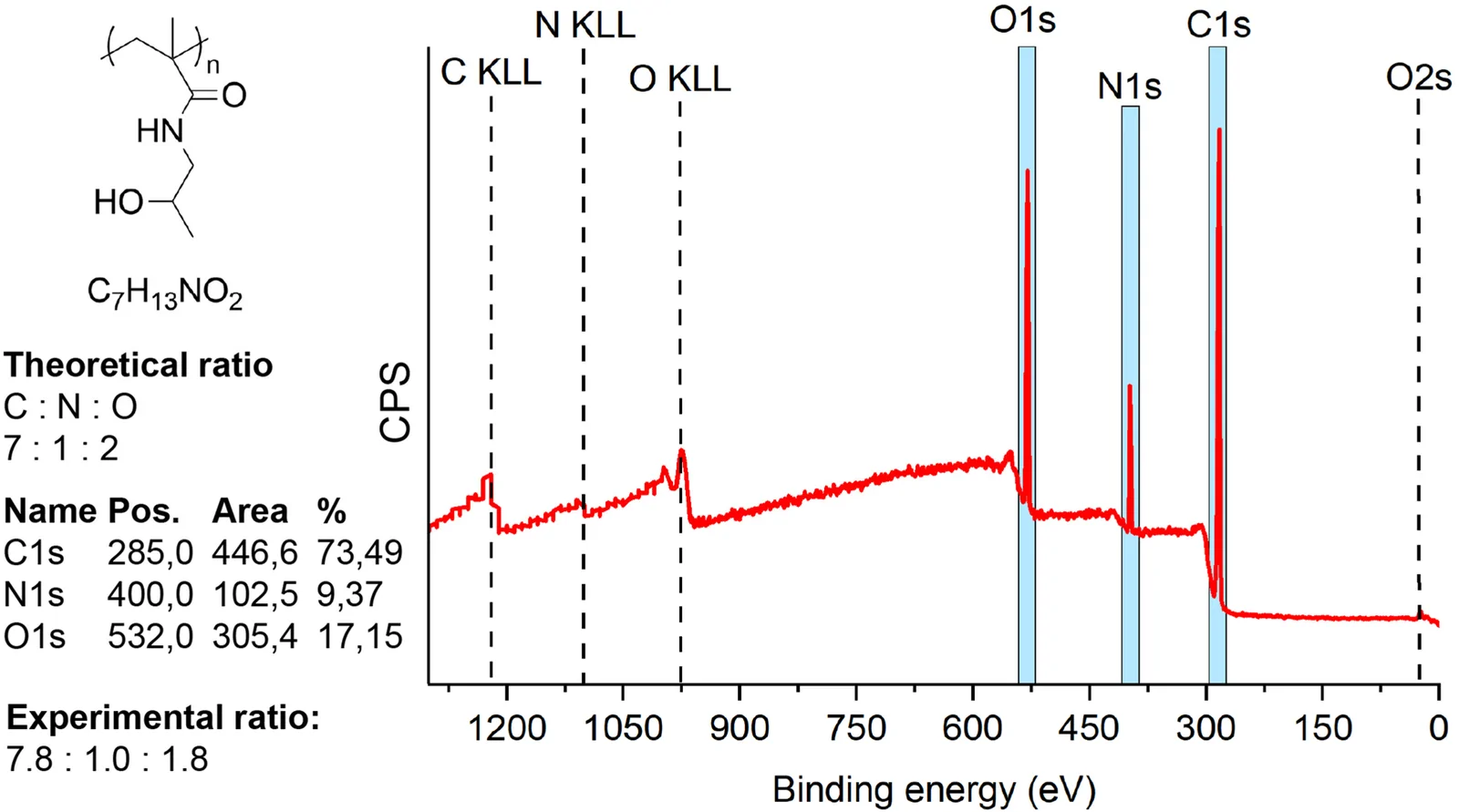
The figure demonstrates the wide-scan XPS spectrum of poly(*N*-(2-hydroxypropyl) methacrylamide) (poly(HPMA)), showing three main peaks: C 1s, N 1s, and O 1s. Adapted from ref. 32 under the terms of the Creative Commons Attribution 4.0 International License (CC BY 4.0). © 2025 The Authors. Published by the American Chemical Society.

For example, consider a polymer brush from *N*-(2-hydroxypropyl) methacrylamide (HPMA) (monomer formula: 7 C, 1 N, 2 O) (Fig. 1). In a well-formed brush >20 nm thick one might expect the C : N : O ratio (ignoring adventitious C) roughly consistent with 7 : 1 : 2. The experimental atomic concentrations (C 73.5%, N 9.4%, O 17.2%) correspond to a C : N : O ratio of 7.8 : 1.0 : 1.8 when normalized to nitrogen, in good agreement with the theoretical 7 : 1 : 2 of the HPMA repeat unit. These ratios are obtained from sensitivity-factor-corrected atomic percentages, not from raw peak areas (see below); comparing raw C 1s, N 1s and O 1s areas directly would give an incorrect composition because each core level has a different photoionization cross-section and is detected with a different analyzer transmission. Occasionally, chain-end functionality (*e.g.*, sulfur or bromine, depending on the method of polymerization: ATRP or RAFT) may appear in the XPS survey, but because such end-groups are sparsely present and may degrade under the X-ray beam, their signals may be weak or undetectable.

When fitting survey spectra, peak-area calculation requires a background subtraction step. Many users default to a linear background, especially when spectra are simple. However, it is worth noting that the literature identifies at least three common background models: linear (straight line), Shirley, and Tougaard, and shows that for wide-range spectra with inelastic scattering or substrate contributions, more sophisticated models (*e.g.*, Tougaard) can produce more accurate results.^46^

Therefore, while a linear background may be acceptable for many polymer-brush survey scans, one should evaluate whether loss features or overlap from the substrate demand a Shirley or Tougaard background. Consistency across all spectra is essential for reliable comparisons.

Atomic concentrations are not obtained from raw peak areas. Each integrated core-level area is first divided by a relative sensitivity factor (RSF) that combines the photoionization cross-section, the analyzer transmission function, and, in most software libraries, an attenuation term; the corrected areas are then normalized to 100%. Because RSF sets differ between instruments and software (*e.g.* Scofield-based libraries in CasaXPS *vs.* Wagner-based sets), the library used should be reported, and compositions from different laboratories should be compared with care. Two further points matter for brushes: (i) hydrogen is not detected by XPS (its photoionization cross-section is effectively zero), so all reported compositions are hydrogen-free and stoichiometric checks must use only the heavier atoms; and (ii) minor elements such as Br, S, P, Si and metals have detection limits of roughly 0.1–1 at%, so sparse chain-end groups may sit near or below the detection limit even when present. Finally, a visible substrate signal can mean either incomplete brush coverage or simply that the film is thinner than the information depth; the two are distinguished by combining the survey with the attenuation analysis above and, where possible, an independent measurement of surface morphology such as AFM. Laterally averaging techniques are less suitable for this particular question: ellipsometry, for instance, averages over a large area, so incomplete coverage and a uniformly thinner film can produce a similar apparent thickness, whereas AFM resolves uncovered regions and defects directly.

When selecting the areas of peaks in wide (survey) spectra, most modern XPS data-processing software (*e.g.*, CasaXPS,^47^ Kratos Vision, Thermo Avantage, RXPSG) will automatically identify and integrate peaks for quantitative analysis. However, the user should always inspect these automatically selected regions, especially when there are nearby peaks, noise, inelastic-scattering backgrounds, or Auger electron features that may overlap with the target signal. Manual adjustment of the integration limits is often required to ensure accurate elemental quantification.

For elements whose core levels are well isolated and symmetric, such as C 1s, N 1s, and O 1s, area selection and quantification are relatively straightforward. However, for elements with p- or d-orbitals, additional care is required because these levels exhibit spin–orbit splitting. For example, the S 2p line is a spin–orbit doublet: S 2p_3_/_2_ and S 2p_1_/_2_, separated by ∼1.18 eV with a fixed 2 : 1 area ratio.^2^ In practice, the doublet is fitted as a single constrained component pair, the splitting and the 2 : 1 ratio held fixed, and quantification uses the summed area of both peaks together with the sensitivity factor defined for the complete S 2p line in the software library. The main risk to quantification is therefore not a different sensitivity factor for each component, but incomplete or inconsistent integration of the doublet, or overlap with a neighboring Auger or substrate feature.

Where the S 2p region overlaps with substrate or Auger features, sulfur can alternatively be quantified from the S 2s line, which appears as a single unsplit peak. S 2s is weaker and offers poorer chemical-state resolution than S 2p, so it is best reserved for total-sulfur quantification rather than for distinguishing oxidation states (for which the resolved S 2p doublet, *e.g.* thioether/RAFT sulfur near 164 eV *versus* sulfonate near 168 eV, remains preferable) (Table 3).

**Table 3:** Doublet constraints for elements common in brushes

Line	Components	Splitting (eV)	Area ratio
S 2p	2p_3/2_ : 2p_1/2_	∼1.18	2 : 1
Si 2p	2p_3/2_ : 2p_1/2_	∼0.6^a^	2 : 1
P 2p	2p_3/2_ : 2p_1/2_	∼0.87	2 : 1
Cl 2p	2p_3/2_ : 2p_1/2_	∼1.6	2 : 1
Br 3d	3d_5/2_ : 3d_3/2_	∼1.05	3 : 2
Au 4f	4f_7/2_ : 4f_5/2_	∼3.67	4 : 3

a Often unresolved.

Wide-range survey XPS spectra can be used to estimate the thickness of polymer-brush films by analyzing the attenuation of substrate photoelectron peaks. In this method, the intensity of a substrate signal, for example, the Au 4f peak on a clean gold surface, is compared to its reduced intensity after polymer-brush deposition, as applied by van Andel and co-authors.^48^ Because the attenuation follows an exponential decay with overlayer thickness, the change in substrate peak intensity can be converted into an “effective XPS thickness” using an assumed electron attenuation length and take-off geometry. The substrate intensity decays exponentially with overlayer thickness: *I* = *I*_0_ exp[−*d*/(*L* cos *θ*)], where *I*_0_ and *I* are the substrate photoelectron intensities before and after brush growth, *d* is the dry overlayer thickness, *L* is the effective attenuation length of the substrate photoelectrons within the brush material, and *θ* is the photoelectron emission angle measured with respect to the surface normal. Rearranging gives the attenuation-equivalent thickness, *d* = *L* cos *θ* ln(*I*_0_/*I*).^82^ Note the angle convention: if the take-off angle *β* is instead defined relative to the sample plane, as some instrument software does, then cos *θ* is replaced by sin *β*. A single *L* value is adequate for emission angles up to ∼60° from the normal; at more grazing angles, *L* itself becomes angle-dependent.^4^ This approach is often appealing when ellipsometric modeling is challenging, such as on gold substrates where strong absorption, plasmonic effects, and surface roughness complicate optical fits. However, XPS-derived thickness values must be interpreted with caution. The method relies on several restrictive assumptions: that the polymer brush forms a uniform, laterally homogeneous, fully covering overlayer; that its density and composition are known; that the electron attenuation length used is appropriate for the actual material; and that the dry morphology under vacuum accurately reflects the true structure of the brush. Polymer brushes frequently deviate from these assumptions; they may have variable density, inhomogeneous coverage, or structural collapse in vacuum, leading the XPS model to return an attenuation-equivalent thickness rather than the real geometric height. Thus, XPS provides a qualitative or comparative measure of film thickness rather than an absolute metric unless the assumptions are rigorously validated. For accurate determination of polymer-brush thickness and morphology, XPS should be complemented with independent techniques such as ellipsometry, AFM, or X-ray/neutron reflectivity, which are sensitive to the actual physical height and structural organization of the brush.^15,83,84^

## C 1s narrow spectra

The C 1s high-resolution (narrow) spectrum is the most commonly used region for the chemical characterization of polymer brush coatings, since these coatings are typically synthesized from carbon-containing monomers. When working with C 1s spectra of polymer brushes, the first step is to choose an appropriate background model. In many cases, a Shirley background is a suitable and convenient option. However, Shirley backgrounds fail when the spectrum contains significant inelastic loss structures, such as loss tails or plasmon peaks.

Although a Shirley background can produce seemingly low residuals and *χ*^2^ values, it may still yield highly inaccurate peak areas and incorrect peak ratios. In some test cases, errors exceeded 50%. In contrast, the Tougaard 4-parameter background generally provides excellent fits by reproducing the correct inelastic scattering shape, peak areas, and peak positions, even for spectra with pronounced loss structures.

For this reason, the improved 4-parameter Tougaard background should be used for:

• wide energy windows,

• spectra with strong background or extended tails,

• materials exhibiting significant inelastic scattering.

A Shirley background should only be used for:

• narrow fitting regions,

• spectra without noticeable loss features,

• cases where the background is weak and flat.

For methacrylate- and methacrylamide-based polymer brush coatings, the Shirley background is typically adequate because these materials usually do not exhibit strong loss tails. For the insulating organic polymers considered here, we use a symmetric Voigt-type line shape with a modest Lorentzian contribution, for example GL(30) in CasaXPS notation, *i.e.*, 30% Lorentzian/70% Gaussian. Line-shape notation is not standardized between packages (GL, SGL and LA parameterize the Gaussian/Lorentzian mix differently), so the exact convention should always be stated. An excessively Lorentzian shape broadens the peak tails and can distort fitted component areas in insulators and should be avoided.

When fitting the C 1s spectrum, it is important to consider how many distinct chemical carbon species exist in the polymer structure. The energy separation between components should generally be at least 0.4 eV; if the separation is smaller, the peaks may not represent distinct states and should potentially be merged. Peaks corresponding to similar bonding environments typically have similar FWHM values. The fitted components are typically constrained to similar full-width at half maximum (FWHM) values, generally in the range of ∼1.2–1.4 eV, although the exact FWHM depends on factors such as analyzer pass energy, instrumental resolution, and sample charging.

**Fig. 2:**
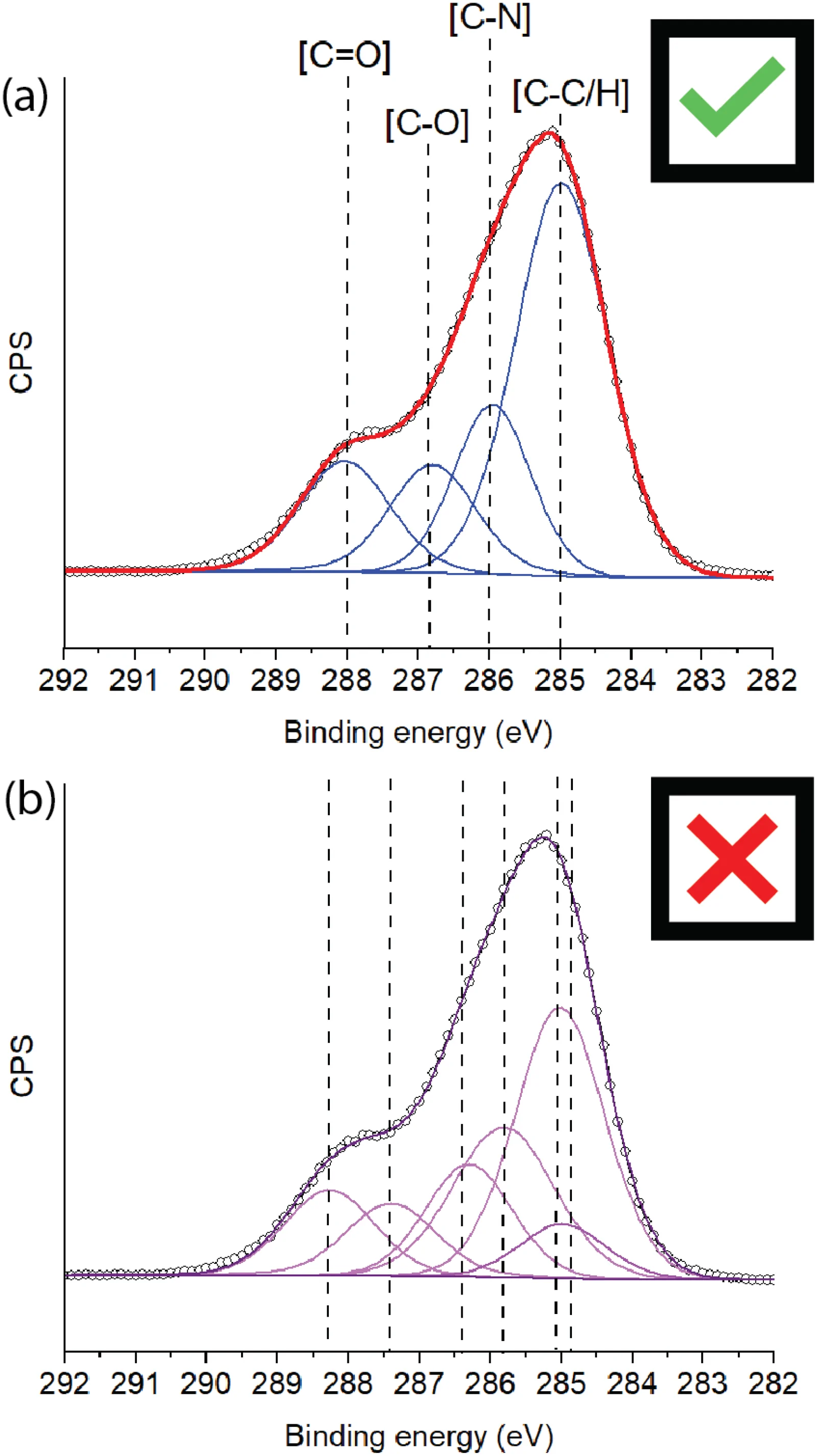
Typical C 1s spectra of poly(HPMA) showing a correct fit (a) and an incorrect fit (b), illustrating common mistakes in peak fitting. Adapted from ref. 32 under the terms of the Creative Commons Attribution 4.0 International License (CC BY 4.0). © 2025 The Authors. Published by the American Chemical Society.

For example, in the case of poly(HPMA), the repeating unit contains four distinct carbon bonding environments: [C–C/C–H], [C–N], [C–O], and [C=O]. The expected ratio between these species is approximately 4 : 1 : 1 : 1 based on the chemical structure. As shown in Fig. 2a, the main C 1s components are observed at binding energies of approximately [C–C/C–H] (285.0 eV), [C–N] (286.1 eV), [C–O] (287.0 eV), and [C=O] (288.0 eV). The fitted peak ratio is 4.7 : 1.3 : 1.0 : 1.0, which is in good agreement with the expected stoichiometric ratio. This fitting also aligns well with simulated XPS spectra based on DFT calculations, which predict a ratio of 4 : 1 : 1 : 1.^32,36,56^ A typical correct fitting is demonstrated in Fig. 2a. In contrast, a common mistake in XPS fitting is shown in Fig. 2b, where the spectrum is overfitted by introducing too many peaks. While this may improve the mathematical quality of the fit, it leads to results that are not physically meaningful or chemically justified. In addition, Monte Carlo error analysis can be used to estimate uncertainties in fitted peak positions, widths, and areas, thereby providing a more realistic assessment of the confidence in quantitative interpretations. Such approaches are increasingly recommended for chemically complex C 1s spectra where overfitting and parameter degeneracy are common.^85^ It is generally good practice to constrain the fitting to chemically reasonable carbon bonding environments, such as [C–C/C–H], [C–N], [C–O], and [C=O], as supported by the molecular structure. If there is uncertainty in peak assignment, DFT calculations of binding energy shifts can provide valuable guidance.

The lowest-binding-energy peak in a C 1s spectrum is usually the [C–C/H] component. This peak is commonly used to reference the spectrum to 285.0 eV. Typical binding energies for various functional groups are listed in Table 4, but these energies may vary (approximately 0.1–0.5 eV) depending on material environment, surface morphology, and local chemistry. Because of such variations, it is often advantageous to perform DFT calculations of the expected binding energies for the monomer units that compose the polymer brush.

**Table 4:** Typical C 1s binding energies by functional group

Carbon bonding type	Approximate C 1s binding energy (eV)
C=C/C–C/H (hydrocarbon)	284.3–285.0
C–N (amine)	285.5–286.2
C–O/C–O–C (ether/alkoxy)	286.0–287.0
C=O (carbonyl)	287.5–288.5
O–C=O (carboxylate/ester)	288.5–289.5
CF, CF_2_, CF_3_ (increasing with F content)	289.0–294.0
Shake-up – π to π*	290.8–291.5

While the preceding sections used poly(HPMA) as a worked example, the same workflow, survey quantification, stoichiometric checks, and constrained C 1s fitting apply directly to other polymer-brush chemistries. Table 5 summarizes the diagnostic XPS signatures of the most common brush classes, including the heteroatom regions that identify each chemistry and the chain-end signals introduced by ATRP and RAFT. The binding energies listed are nominal values referenced to C–C/C–H at 285.0 eV; exact positions vary with instrument, charging behavior and referencing choice, and should be treated as starting points for fitting rather than fixed constraints.

**Table 5:** Diagnostic XPS signatures for common brush chemistries^86^

Brush type	Key diagnostic signatures
PEG/oligo(ethylene glycol) (OEGMA, MEO_2_MA)^38,40,56,87^	Dominant C–O ether component ∼286.5; high C–O : C–C/H ratio; ether O 1s ∼532.8; no nitrogen
Zwitterionic^48,59,88,89^ carboxybetaine (CBMA)	Quaternary-ammonium N 1s ∼402.3; carboxylate O–C=O ∼288.5; N present with characteristic C : N of the betaine unit
Zwitterionic sulfobetaine (SBMA)	R_4_N^+^ N 1s ∼402.3; sulfonate S 2p (S^6+^) ∼167–168—clearly separated from thioether/RAFT S ∼164
Cationic/quaternized^90^ (PDMAEMA, quaternized)	Amine N 1s ∼399.5 *vs.* protonated/quaternary N ∼401.5–402.5; strongly beam-sensitive
Acrylate/methacrylate^91^ (PMMA)	Ester O–C=O ∼289.0 and C–O ∼286.7 in ∼1 : 1; no nitrogen
Hydroxy-methacrylate (PHEMA)	C–O ∼286.5 and O–C=O ∼289.0; hydroxyl contribution in O 1s
Fluorinated (PFMA, fluoroacrylate)^92^	CF_2_ ∼291, CF_3_ ∼293.5; strong F 1s ∼688–689; large F sensitivity factor
Aromatic/polystyrene^93–95^ (PS)	π → π* shake-up satellite ∼291.5 (∼6–7 eV above the main C–C line); otherwise a single symmetric C 1s

## XPS depth profiling of polymer brushes

Depth profiling of polymer brushes by XPS is most commonly performed using argon gas cluster ion beams (GCIB) rather than monatomic Ar^+^ sputtering, although older instruments are often equipped only with Ar^+^ sources.^80^ Monatomic sputtering typically induces severe chemical damage in organic materials, including cross-linking, carbonization, and preferential sputtering. In contrast, cluster sources (*e.g.*, Ar_*n*_^+^ with *n* ≈ 500–2000 atoms per cluster) distribute the impact energy over many atoms, thereby reducing subsurface damage and enabling near-steady-state sputtering in many polymers.^80^ Systematic studies have shown that sputter behavior depends strongly on whether the polymer is “self-sealing” (cross-linking dominated, type I) or “self-opening” (bond-scission dominated, type II) under irradiation. In the latter case, depth profiling is generally more reliable, as scission events maintain sputter yield with depth, whereas cross-linking can suppress sputtering and degrade depth resolution.^96^

However, even when using GCIB, depth profiling of polymers is not purely ion-controlled. A critical observation reported by Cumpson and co-workers^49^ is that X-ray irradiation itself can significantly modify sputter rates during profiling, producing additional craters within the ion-beam crater. In some degrading polymers (*e.g.*, PLLA and PCTFE), X-ray exposure has been shown to increase the effective sputter depth by up to a factor of three. As a result, the crater bottom is not ideally flat, but may instead exhibit a U-shaped or centrally deepened morphology. This additional topography directly reduces depth resolution and complicates thickness calibration, particularly for nanometer-scale polymer brushes where the total layer thickness may be comparable to the X-ray-assisted crater depth.

To minimize X-ray-induced sputter enhancement during GCIB depth profiling, the X-ray beam should ideally be blanked, shuttered, or moved away from the analysis region during sputtering intervals when spectra are not actively being acquired.^49^ This approach reduces the cumulative radiation dose delivered to the polymer and helps preserve a flatter crater geometry and more reliable depth resolution. Such precautions are particularly important for radiation-sensitive polymers and ultrathin polymer-brush architectures, where even modest X-ray-induced changes may significantly affect the apparent depth profile.

Within these constraints, depth profiling can still be used to confirm the chemical composition and layering of polymer brushes. For example, depth profiling was used to confirm the diblock structure of poly(HPMA)-poly(MeOEGMA) brushes prepared by SET-LRP, by monitoring changes in the C 1s spectra after successive sputtering intervals.^62^ Similarly, the diblock structure of poly(HPMA)-poly(CBMA) brushes prepared by SI-PET-RAFT was corroborated using the same approach.^60^

Several studies demonstrate that cluster-ion depth profiling, using argon gas cluster ion beams (GCIB) or, in earlier work, C_60_^+^ fullerene sputtering, can provide compelling evidence for hierarchical or layered brush architectures (C_60_^+^ is a molecular fullerene projectile and is not an argon gas cluster ion beam: although both are polyatomic and far gentler than monatomic Ar^+^, they differ in projectile mass, energy per atom, and damage behavior, and results from the two should not be treated as equivalent). For example, Rodriguez-Emmenegger and co-workers used XPS depth profiling to confirm a stacked diblock brush poly(HPMA-*b*-MeOEGMA), observing a clear transition in C 1s component ratios with depth consistent with enrichment of the HPMA block deeper in the film.^62^ Similarly, diblock and random antifouling/bioactive brush platforms were investigated, prepared by SI-PET-RAFT using HPMA and CBMA, and XPS depth profiling was used to corroborate the intended layered compositions.^60^ Further examples include explicitly stacked hierarchical brushes such as PHEMA (15 nm)-*b*-PS(40 nm) and PHEMA (15 nm)-*b*-PEtHexMA (54 nm), where GCIB depth profiling of the C 1s region progressively revealed the underlying PHEMA contributions as the top block was removed.^97^

Depth profiling is also useful for probing functional gradients within a single brush. Barbey and coauthors combined XPS with C_60_ sputtering to show that small post-modifiers (*e.g.*, propylamine) can penetrate throughout PGMA brushes, whereas larger biomolecules (*e.g.*, BSA) remain predominantly localized near the surface.^19^

In summary, GCIB depth profiling is a powerful tool for investigating polymer brushes, including diblock architectures. However, its quantitative reliability depends critically on controlling and reporting crater geometry, X-ray overlap, and potential X-ray-induced sputter enhancement. For brushes in which compositional gradients span only a few nanometers, even modest crater non-uniformity can dominate the apparent depth resolution.

To make a depth profile interpretable and reproducible, the following should be reported: cluster size and energy (hence energy per atom, in eV per atom), the sputter raster area and the analysis area within it, the sputter interval per cycle, and the method used to measure the final crater depth (*e.g.* stylus profilometry or AFM) for depth calibration. Even under GCIB, interface broadening and some preferential sputtering or chemical modification remain, so a monotonic change in a C 1s component ratio with sputter time is best described as a compositional trend rather than a calibrated concentration depth profile unless the crater depth has been measured independently.

## DFT calculations and predictions of XPS spectra

Density functional theory (DFT) calculations can be used to calculate the absolute binding energy of core electrons in atoms. These binding energies vary slightly (of the order of a few eV) when the atom is bonded in different chemical environments.^98^ By assigning carbon atoms (or other elements) to their characteristic binding-energy ranges, DFT helps identify the number and type of species expected in the XPS spectrum and can be used to generate or validate predicted spectral components.^32,56,99–102^

Computational chemistry is often synergistic with spectroscopy. It provides both an aid for interpretation and access to otherwise inaccessible information. Several quantum chemical methods exist to study X-ray photoabsorption processes, including coupled-cluster response theory, which has been successfully applied in this context.^103^

For X-ray photoelectron spectroscopy (XPS), a relatively simple ΔSCF approach can be employed within wavefunction theory or density functional theory. In this approach, the emitted electron is explicitly removed from a core level. The ΔSCF procedure involves two separate self-consistent field (SCF) calculations: one for the reference (neutral) molecule and one for the core-ionized species.^104^ The difference in total energy between these two states is taken as the binding energy of the emitted core electron. In an SCF procedure, all occupied molecular orbitals are optimized to minimize the total energy. Hence, the calculated binding energy is sensitive to the chemical environment of the core-ionized atom. Within an effective one-electron potential scheme, as used in DFT, core ionization reduces the shielding of nuclear charge experienced by the remaining electrons. As a result, all other electron levels shift to lower orbital energies, affecting interelectronic interactions throughout the system. This effect propagates up to the valence levels, influencing hybridization and bonding. The total energy of the core-ionized state, therefore, reflects the collective response of all electrons to the presence of the core hole. Consequently, the ΔSCF-computed core electron binding energy is sensitive to the local bonding environment of the ionized atom.

When comparing with the experiment, several factors must be considered. A ΔSCF approach provides binding energies but cannot yield spectral intensities; however, counting the number of equivalently bonded atoms can yield an intuitive estimate of relative peak intensities. In XPS, the kinetic energy of the emitted electron is measured and related to the energy of the incident X-ray photon. Surface potentials and other experimental effects that may shift the spectrum are not explicitly accounted for in standard calculations.

**Fig. 3:**
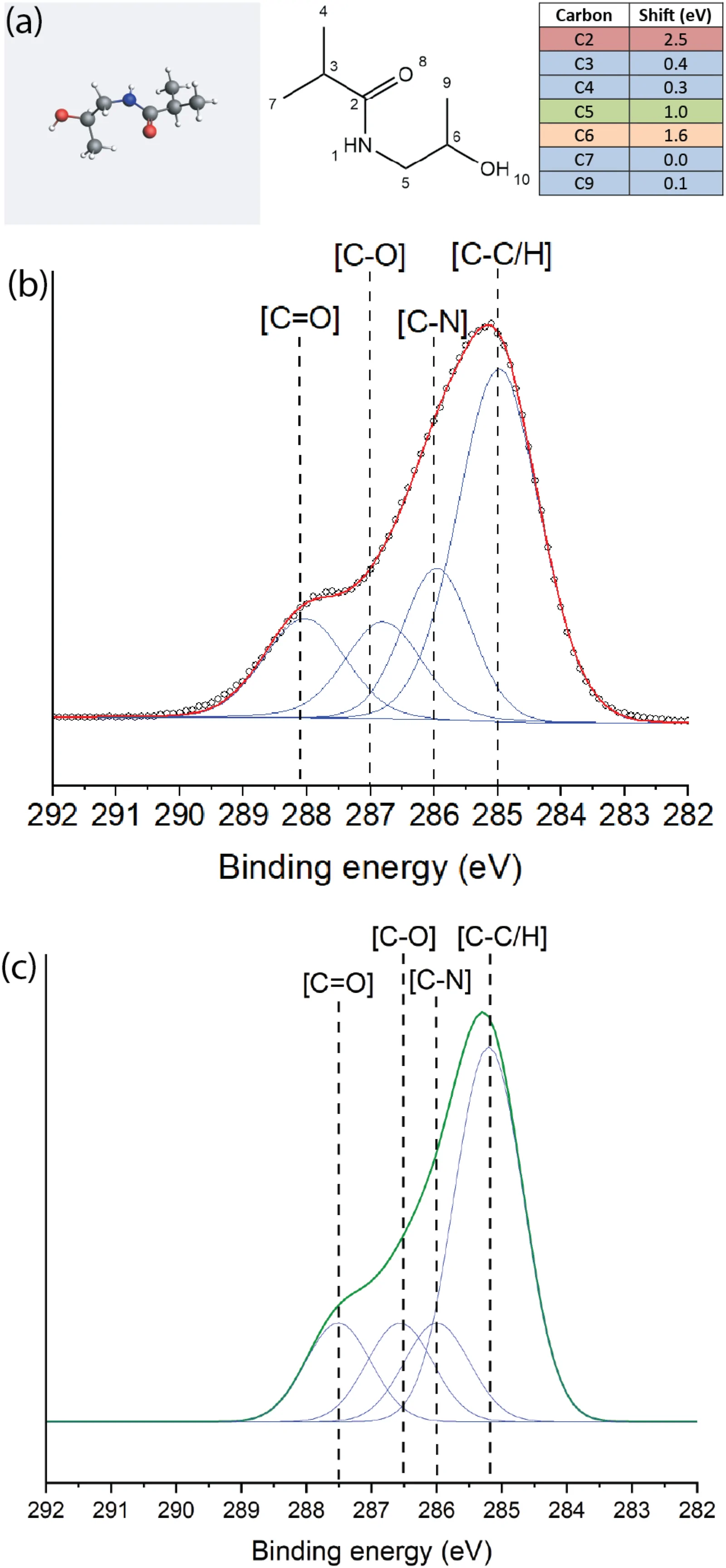
The figure shows (a) the HPMA unit with DFT-calculated energy shifts, (b) the measured C 1s spectrum, and (c) the simulated spectrum based on DFT-calculated energy shifts. Adapted from ref. 32 under the terms of the Creative Commons Attribution 4.0 International License (CC BY 4.0). © 2025 The Authors. Published by the American Chemical Society.

Experimental C 1s XPS spectra are often calibrated by aligning the C 1s peak to approximately 284–285 eV (the exact value depends on the convention used). A similar alignment can therefore be applied to computed spectra to enable meaningful comparison of relative chemical shifts of the binding energy. For example, as shown in Fig. 3, the calculated relative binding energy shifts can be used to group carbon atoms, thereby generating a simulated spectrum. Density functional theory (DFT) calculations can therefore be used not only to predict binding energies but also to support the assignment of spectral features by correlating computed shifts with chemical environments.

### Computational protocol

A simulated XPS spectrum can be obtained using DFT calculations to obtain core-electron binding energies, and using a pragmatic estimate for the XPS signal amplitude. DFT calculations can be performed using the Amsterdam Density Functional (ADF) computational chemistry software, part of the Amsterdam Modeling Suite (AMS), or using other software such as Gaussian.^105^ We follow computational protocols developed for X-ray absorption spectrum (XAS) calculations on oligothiophenes, the BP86 generalized gradient approximation (GGA) functional^106,107^ together with a TZ2P basis set as implemented in the Amsterdam Density Functional (ADF) package.^105^ A small frozen-core approximation is used, in which chemically inactive core orbitals are kept fixed during the self-consistent field procedure to reduce computational cost.^105^ Scalar relativistic effects were included using the zero-order regular approximation (ZORA).^108,109^ The start of the computational protocol is a geometry optimization of the neutral molecule complemented by a vibrational frequencies calculation to check whether a global minimum was reached on the potential energy surface: a vital computational prerequisite. Here, we chose the monomer as a model system for the surface-attached polymer, but in principle other choices can also be made, such as a capped monomer, a surface-bound fragment, and so on. To obtain the carbon 1s (C 1s) core-electron binding energy using the ΔSCF (*vide supra*) method, the total energies for the neutral ground state and core-ionized state need to be calculated. For the neutral molecule, one carbon atom is selected for core-ionization in the next calculation. On this atom alone, an all-electron basis set should be configured, and the spin-unrestricted formalism should be used. The total energy (total bonding energy as reported in AMS), *E*_neutral_, serves as the reference energy for the core-ionization energy calculation. The spin-unrestricted SCF orbitals obtained for the neutral molecule allow the core hole to be created at a carbon atom of choice, and its all-electron basis on that atom prevents nonlocal behavior. From the orbital lowest in energy, the core electron is removed by setting the occupation of the beta spin orbital to zero. Calculation of the energy *E*_hole_ allows the calculation of the binding energy (BE) of the C 1s electron, BE(C 1s) = *E*_hole_ − *E*_neutral_. The direct chemical environment of the carbon atom used has an influence on *E*_hole_ and thereby influences BE(C 1s). By performing a core-electron binding energy calculation of each carbon atom, all peak energies in the XPS spectrum are obtained. We choose to perform our calculations on only the chemically distinct carbon atoms, and we used the number of chemically equivalent carbon atoms in the molecule as a linear measure of peak height. Peak broadening can be achieved by plotting a Gaussian function with a suitable width. The total XPS spectrum is obtained by adding all Gaussians.

## Conclusions

In this tutorial, we have outlined practical guidelines for the reliable application of X-ray photoelectron spectroscopy (XPS) to polymer brush coatings. Owing to its inherent surface sensitivity, XPS is particularly well suited for characterizing these thin, nanometer-scale films, but careful experimental design and data analysis are essential to obtain meaningful results. Key considerations include proper sample preparation, control of contamination and beam-induced damage, and consistent reporting of acquisition parameters to ensure reproducibility.

The interpretation of both survey and high-resolution spectra requires a chemically informed approach. In particular, C 1s peak fitting should be constrained by the known molecular structure, expected stoichiometry, and reasonable energy separations between components. Overfitting may improve statistical agreement but can lead to physically incorrect conclusions. Depth profiling using GCIB provides valuable insight into layered and gradient structures in polymer brushes, although its quantitative reliability depends on understanding sputtering artifacts and X-ray-induced effects.

Finally, the integration of density functional theory (DFT) calculations offers a powerful complementary tool for interpreting XPS data. By predicting relative binding energy shifts, DFT can guide peak assignment and support chemically consistent fitting, particularly in complex systems. When combined with careful experimental practice and critical data interpretation, XPS remains a robust and versatile technique for investigating the composition and structure of polymer brush coatings.

Looking ahead, several developments are likely to reshape XPS of polymer brushes. Near-ambient-pressure and *in situ*/*operando* configurations are beginning to allow surfaces to be probed closer to their solvated, functional state rather than only in the dry, collapsed state imposed by ultrahigh vacuum.^110,111^ Such measurements have so far been applied mainly to oxide and electrocatalytic interfaces, but the recent availability of laboratory-based *in situ* and *operando* instrumentation makes their extension to hydrated polymer-brush systems a realistic prospect. On the analysis side, Monte-Carlo error estimation and emerging machine-learning approaches offer a route to more objective and reproducible spectral decomposition. Convolutional neural networks trained on large synthetic spectral libraries have been shown to identify and quantify elemental composition with an accuracy comparable to that of experienced analysts,^112^ and related frameworks now add uncertainty estimation to automated chemical-state quantification.^113^ Extending such methods to the C 1s envelopes typical of polymer brushes, where component overlap and charge referencing are the dominant difficulties, is a natural next step. Finally, correlative characterization, combining XPS with ellipsometry, AFM, neutron or X-ray reflectivity, and ToF-SIMS, remains the most reliable way to convert the surface-sensitive, composition-rich but depth-limited XPS signal into a complete picture of brush thickness, density profile, and buried-interface chemistry. Together with the consistent acquisition and transparent reporting emphasized throughout this tutorial, these directions should make XPS characterization of polymer brushes both more physically faithful and more reproducible.

## Conflicts of interest

There are no conflicts of interest to declare.

## Data Availability

No primary research results, software, or code have been included, and no new data were generated or analyzed as part of this tutorial article. All illustrative data discussed in the article are derived from previously published sources cited in the manuscript.
